# Association of anxiety and depression to headache, abdominal- and musculoskeletal pain in children

**DOI:** 10.3389/fpain.2023.1136145

**Published:** 2023-04-12

**Authors:** Marianne Nilsen, Siri Weider, Marte Kathrine Halse, Charlotte Fiskum, Lars Wichstrøm

**Affiliations:** ^1^ Department of Social Work, Faculty of Social and Educational Sciences, NTNU, Norwegian University of Science and Technology, Trondheim, Norway; ^2^ Department of Psychology, Faculty of Social and Educational Sciences, NTNU, Norwegian University of Science and Technology, Trondheim, Norway; ^3^ Department of Child and Adolescent Mental Health, St. Olav’s Hospital, Trondheim, Norway

**Keywords:** recurrent pain, anxiety, depression, children, headache, abdominal pain, muculoskeletal pain

## Abstract

The comorbidity between recurrent pain, anxiety, and depression among children is frequent and well documented. However, only a few studies of the predictive effect of anxiety and depression on pain have adjusted for symptoms of the other disorder when examining the respective relations to different pain locations, rendering the unique contribution from anxiety and depression undetermined. In the current investigation we explore the strength of associations between pain at different locations with symptoms of anxiety and depression in a community sample of 10-year-old children (*n* = 703). The children were interviewed about the frequency of pain during the last 3 months. Parents and children were interviewed separately about symptoms of anxiety and depression using a semi-structured diagnostic interview. Results of three multivariate regression models for each of headache, abdominal and musculoskeletal pain revealed that depression was associated with musculoskeletal pain and headache, whereas anxiety was not. The associations for depression were not significantly stronger compared to anxiety. Gender-specific models found that depression was related to headache only among girls, but the association was not statistically different compared to boys. These results may, in turn, influence our interpretation of different forms of pain in children, with less weight given to abdominal symptoms viewed as a strong correlate with psychological problems, compared to for instance headache. The results provided no clear support for neither a differential relationship between anxiety and pain and depression and pain nor gender differences.

## 1. Introduction

Recurrent pain is prevalent in children and adolescents (i.e., 11%–40%) (1, 2), and is linked to challenges in other areas of life such as disruption of education, increased school absence, and difficulties forming friendships (3–8), as well as socioeconomic difficulties (9). Recurrent pain has a multifactorial etiology (1), and may result from environmental adversities and mental health problems (10–15). Thus, the comorbidity between pain and a range of psychiatric disorders and symptoms (15, 16), especially anxiety and depression (1, 2, 13, 15, 17–20) is well documented and common. Furthermore, the association between pain and internalizing symptoms is part of an understanding of how children often present symptoms of psychopathology as somatic rather than psychological discomfort (21).

Notably, anxiety and depression are also highly comorbid with each other (22). Odds ratio for concurrent comorbidity of anxiety and depression among 9–16 year olds in a community sample is reported to be 28 for boys and 29 for girls (23), when also controlling for comorbidity with behavioral disorders. Therefore, before attempting to understand the reasons for the comorbidity between pain and anxiety and depression respectively, we need to examine whether these comorbidities (i.e., anxiety and pain; depression and pain) are independent, or if one of the comorbidities is driving the other. At present, such research is lacking. Although longitudinal studies are needed to illuminate the temporal relationship between anxiety/depression and pain, cross-sectional studies can determine the unique association between anxiety and pain, and between depression and pain, which is the main aim of the current study.

The relation between negative emotions and pain is complex, and is likely moderated or mediated by a range of factors (e.g., gender, catastrophizing, coping skills, hopelessness, social support, interpersonal relationships, self-efficacy, somatization) (24). These factors may be differentially related to the anxiety-pain and the depression-pain associations, as seen with coping skills in one community sample of children aged 8–15 years; higher level of active coping was related to less pain *via* fewer symptoms of depression (but not anxiety) (25). Although anxiety and depression may be linked to pain through a variety of mechanisms, few studies have adjusted for symptoms of anxiety when examining associations between depression and different pain locations in community samples of children. Nevertheless, when both anxiety and depression have been controlled for in community samples, results have revealed that girls with depressive disorders reported both more frequent headaches and more severe effects of headache compared to girls with anxiety disorders (no associations were found for boys) (26, 27). Furthermore, for girls, a combination of stomachaches and headache—and musculoskeletal pains alone were related to anxiety disorder (27). Musculoskeletal pain alone was also associated with depression for both boys and girls (27). These findings suggest that anxiety and depression may differ in their association to pain—dependent upon where the pain is located, as well as gender. The above-mentioned studies did, however, not specifically test whether the associations between anxiety and depression with pain were significantly different (26, 27).

As the number of empirical studies addressing the relationship between pain, anxiety and depression in children are few, the current study is explorative. We aim to examine the strength of associations between pain at different locations with symptoms of anxiety and depression in a community sample. As few studies have controlled for symptoms of anxiety when studying depression, and vice versa, we do so in the current study allowing us to determine the unique contribution of symptoms of anxiety and depression in association to pain. In addition, we extend previous work by explicitly testing for significant differences between the unique contribution of anxiety and depression to pain at different localizations by utilizing data from a community study of 10-year-olds, and inquire: (1) Are anxiety and/or depression associated with recurrent headache, abdominal- and musculoskeletal pain? (2) Are there significant differences in the unique contributions from anxiety and depression to pain in different locations (i.e., headache, abdominal- and musculoskeletal pain)? (3) Do the associations of the three pain locations to anxiety and depression differ significantly for boys and girls?

## 2. Method

### 2.1. Procedure and sample

The Trondheim Early Secure Study (TESS) (28) commenced in 2007 when the participating children were 4 years old. The work presented herein uses data from the fourth wave of data collection (child age = 10), when pain was included in the study. The 2003 and 2004 birth cohorts, and their parents living in Trondheim, Norway, were invited to participate in the study. The children were recruited at the community health checkup for 4-year-olds, which is a free service to all Norwegian children. A letter of invitation was sent to all parents (*N* = 3,456) prior to meeting at the well-child clinic. Of these, a total of 3,358 (97%) parents met at the clinic. At the checkup they were informed about the study by the health nurse and written consent to participate was obtained. Parents (*n* = 176) who lacked proficiency in Norwegian language where excluded. The health nurses failed to ask 166 parents. A total of 2,475 of 3,016 eligible parents consented to participate. To increase the study's variability and thus its statistical power, the researchers oversampled children with emotional or behavioral problems. This was done because most children do not typically score high on measures of anxiety and depression, but emotional symptoms are known to be correlated with pain. Therefore, oversampling was necessary in order to maintain variability in both emotional problems and pain. To accomplish this, parents completed the Strengths and Difficulties Questionnaire (SDQ) (29). The SDQ total difficulties scores were divided into four strata (cut offs: 0–4, 5–8, 9–11, 12–40). The higher the scores on the SDQ, the more likely the child was to be drawn to participate in the study. The drawing probability for each SDQ strata was 0.37, 0.48, 0.70, and 0.89, respectively. This oversampling was adjusted for in the analyses. Details concerning the procedure and recruitment are further described in Steinsbekk and Wichstrøm (28). As a result of the procedures described above, 1,250 families were randomly drawn to participate, of which 1,007 (81%) were examined at the first wave. Those who dropped out at this time point did not vary by SDQ strata (*χ*² = 5.70, df = 3, *p* = .13) or gender (*χ*² = .23, df = 1, *p* = .63). At the second wave, 2 years later, 795 children (50.5% boys) participated in the follow-up assessment. Four and 6 years later, in the third and fourth wave, 699 and 703 children participated, respectively. The current study is based on data from the fourth wave. Included in the total sample was *n* = 703, who had responded on at least one variable included in the final model; pain location, anxiety, depression, gender, or socioeconomic status. Of the 703 children, 3 girls had reached menarche by the time of T4 data collection (10 years), and 1 of these 3 girls reported pain during the last 3 months (i.e., “daily musculoskeletal pain”). Sample characteristics are displayed in Table 1. The project has been approved by the Regional Committee for Research Ethics, Mid-Norway.

**Table 1:** Sample characteristics.

	*n* (Total *n*)	Valid %
Gender		
Boys	337 (673)	50
Anxiety		
≤1 Symptom	495 (702)	71
2–3 Symptoms	137 (702)	20
>4 Symptoms	70 (702)	9
Depression		
≤1 Symptom	546 (629)	86
2–3 Symptoms	75 (629)	12
>4 Symptoms	8 (629)	2
Musculoskeletal pain		
<Monthly	522 (601)	87
1–3 times pr. month	26 (601)	4
Weekly	13 (601)	2
2–6 times pr. week	21 (601)	4
Every day	19 (601)	3
Headache		
<Monthly	513 (569)	90
1–3 times pr. month	25 (569)	4
Weekly	10 (569)	2
2–6 times pr. week	15 (569)	3
Every day	6 (569)	1
Abdominal pain		
<Monthly	523 (550)	95
1–3 times pr. month	13 (550)	2
Weekly	3 (550)	1
2–6 times pr. week	7 (550)	1
Every day	4 (550)	1
Ethnic origin of biological mother		
Norwegian	623 (673)	93
Ethnic origin of biological father		
Norwegian	604 (670)	90
Parental socioeconomic status		
Leader	147 (696)	21
Professional, higher level	281 (696)	40
Professional, lower level	175 (696)	25
Skilled workers	89 (696)	13
Farmers/fishermen	1 (696)	0.1
Unskilled workers	3 (696)	0.4

### 2.2. Measures

#### 2.2.1. Demographics

We used The International Standard Classification of Occupations (ISCO) (26) to measure parental socio-economic status (SES). The level of occupational status ranged from 1 (low) to 6 (high); (1) unskilled workers, (2) farmers/fishermen, (3) skilled workers, (4) lower professionals, (5) higher professionals, and (6) leaders. SES was operationalized as the higher occupational status of the two parents when there were two parents. The child’s gender was coded (1) for boys and (2) for girls.

#### 2.2.2. Frequency of recurrent pain

The frequency of recurrent pain was assessed *via* child report. The children were interviewed and asked: “During the past 3 months, how often have you felt pain in the following locations?”: (a) head, (b) abdomen, (c) neck/shoulders, (d) back, (e) arms, (f) legs, (g) other locations. Musculoskeletal pain comprises neck/shoulders, back, arms and legs. The children’s responses were recorded on a seven-point scale with the following options: (1) no pain during the last 3 months, (2) never/seldom during the last 3 months, (3) monthly, (4) 2–3 times a month, (5) weekly, (6) 2–6 times a week, (7) every day. If the child responded (2)–(7), and they reported their pain to be caused by injury or illness, they were recoded as (1) no pain during the last 3 months. For each of the pain localizations, the number of children who reported their cause of pain to be either injury or illness were as follows: headache *n* = 24; abdominal pain *n* = 20; neck/shoulder pain *n* = 6; backpain *n* = 6; pain in the arms *n* = 7; pain in the legs *n* = 53; and pain in other locations *n* = 11.

#### 2.2.3. Anxiety and depression

Symptoms of anxiety and depression were recorded using the semi-structured diagnostic interview Child and Adolescent Psychiatric Assessment (CAPA) (30). The CAPA is developed for assessing mental disorders according to the Diagnostic and Statistical Manual of Mental Disorders, 4th ed. [DSM-IV-TR (31)]. The child and the one parent who came with the child to the study were interviewed separately. The CAPA contains a structured protocol with mandatory questions and optional follow-up questions. A symptom is considered present if reported by either child or parent. Symptom count of (i) major depressive disorder and (ii) separation anxiety, social anxiety, generalized anxiety and the number of specific phobias were created to measure depression and anxiety, respectively. The interviewers (*n* = 7) had at least a bachelor’s degree in a relevant field and were trained by the CAPA team. Blinded raters recoded 15% of the interviews and the resulting intra-rater reliabilities ICC was .87 for symptoms of depression and .86 for anxiety disorders.

### 2.3. Statistical analysis

Correlations between all variables were assessed by Pearson’s *r*. Pearson’s *r* were interpreted in accordance with suggestions that correlations of .10 be considered weak, .20 relatively typical and above .30 fairly strong to strong in psychological studies (32, 33). For each of the three outcomes of headache, abdominal pain, and musculoskeletal pain, a multivariate regression model within a structural equation framework was applied to evaluate whether the anxiety-pain association differed from the depression-pain association. The three recurrent pain locations were regressed on symptoms of depression, anxiety, gender and SES in separate models. First, one model was run separately for the two predictors of anxiety and depression. Next, anxiety and depression were both added as predictors before gender and SES were included in the three models. By means of the Satorra-Bentler’s scaled chi-square test (34) we tested for significant differences between two different models; one model defined anxiety and depression to be equal in correlation to each of the three outcomes (i.e., headache, abdominal pain, musculoskeletal pain), while in the other model anxiety and depression were defined as unequal correlators to the three pain outcomes. Finally, to detect differences across gender, we carried out multiple group analysis in Mplus.

As the counts of symptoms of anxiety and depression were skewed to the right, we used a robust maximum likelihood estimator that handles moderate deviations from normality well (35). Because children with mental health problems were oversampled, population weights were applied using a factor corresponding to the number of children in the stratum divided by the number of participating children. Missing data were handled according to a full information maximum likelihood procedure. Analyses were performed in Mplus 8.0 (36).

## 3. Results

### 3.1. Descriptive findings

Table 2 indicates that frequencies of pain in the various locations (i.e., headache, abdominal pain, and musculoskeletal pain) were strongly correlated with each other. Musculoskeletal pain was weakly related to both anxiety and depression, whereas headache was weakly related to depression only. Abdominal pain did not correlate with neither anxiety nor depression.

**Table 2:** Mean, standard deviations and correlations of study variables for the total sample and boys and girls separate.

	Mean (SD)			Correlations					
	Total sample	Boys	Girls	1	2	3	4	5	6
1. Musculoskeletal pain	1.67 (1.52)	1.61 (1.48)	1.71 (1.52)	-	.79*** (.77***)	.76*** (.76***)	.12* (.10)	.13* (.11)	**.02** (.04)
2. Headache	1.47 (1.21)	1.40 (1.07)	1.53 (1.32)	.79*** (.84***)	-	.80*** (.75***)	**.06** (.08)	.16** (.18*)	**−.01** (.00)
3. Abdominal pain	1.32 (0.92)	1.21 (0.60)	1.42 (1.13)	.76*** (.82***)	.80*** (.89***)	-	**.02** (.00)	**.07** (.03)	**−.07** (−.07)
4. Anxiety	1.10 (1.47)	1.17 (1.50)	1.03 (1.40)	.12* (.10)	**.06** (.03)	**.02** (.07)	-	.45*** (.48***)	**−.01** (.01)
5. Depression	0.53 (0.90)	0.43 (0.72)	0.61 (1.02)	.13* (.13)	.16** (.10)	**.07** (.10)	.45*** (.38***)	-	**−.05** (−.11)
6. SES	4.74 (0.94)	4.75 (0.91)	4.74 (0.97)	**.02** (.00)	**−.01** (−.03)	**−.07** (−.06)	**−.01** (−.01)	**−.05** (−.02)	-

* = *p* < .05, ** = *p* < .01, *** = *p* < .001. Total sample *n* = 703; Boys *n* = 337; Girls *n* = 366. Correlations for girls are displayed in paranthesese on the top diagonal, and for boys in parantheses in the bottom diagonal.

### 3.2. Differential influence of anxiety and depression on pain

The associations between depression and anxiety and each of the three pain locations (i.e., musculoskeletal pain, headache, abdominal pain) showed different patterns (Table 3). More symptoms of both depression and anxiety were associated with more musculoskeletal pain, but the associations with both disorders were attenuated when examined simultaneously (Model 3, Table 3). Depression was related to musculoskeletal pain and headache, even when adjusting for anxiety, gender and SES. Neither anxiety nor depression were related to abdominal pain. The Satorra-Bentler revealed no significant differences between anxiety and depression in their association with the different pain locations; musculoskeletal pain (Δ*χ*^2^ = 1.68, df = 1, *p* = 0.195), headache (Δ*χ*^2^ = 3.65, df = 1, *p* = 0.056) and abdominal pain (Δ*χ*^2^ = 1.69, df = 1, *p* = 0.194).

**Table 3:** Results from regression analysis of the estimated effects of depression, anxiety, gender and socio-economic status (SES) on musculoskeletal pain, headache and abdominal pain.

	Musculoskeletal pain			Headache			Abdominal pain		
	*β*	95% CI	*p*-value	*β*	95% CI	*p*-value	*β*	95% CI	*p*-value
**Model 1**									
Depression	.14	0.012 to 0.261	**.** **032**	.17	0.034 to 0.296	**.** **014**	.06	−0.048 to 0.175	.266
**Model 2**									
Anxiety	.12	0.012 to 0.221	**.** **029**	.05	−0.018 to 0.135	.136	.02	−0.056 to 0.098	.589
**Model 3**									
Depression	.10	−0.030 to 0.235	.130	.18	0.030 to 0.331	**.** **019**	.06	−0.065 to 0.188	.340
Anxiety	.07	−0.047 to 0.185	.245	−.03	−0.126 to 0.065	.530	<.00	−0.087 to 0.094	.934
**Model 4**									
Depression	.09	−0.040 to 0.225	.173	.17	0.022 to 0.320	**.** **024**	.04	−0.083 to 0.174	.490
Anxiety	.06	−0.054 to 0.168	.314	−.03	−0.121 to 0.068	.587	.01	−0.078 to 0.101	.802
Gender	.06	−0.030 to 0.150	.191	.05	−0.035 to 0.139	.242	.10	0.018 to 0.186	**.** **017**
**Model 5**									
Depression	.13	0.012 to 0.257	**.** **032**	.15	0.021 to 0.276	**.** **022**	.12	−0.015 to 0.245	.082
Anxiety	.03	−0.070 to 0.139	.522	−.03	−0.113 to 0.062	.568	.00	−0.086 to 0.089	.971
Gender	.03	−0.059 to 0.110	.554	.06	−0.026 to 0.136	.185	.08	0.005 to 0.163	**.** **037**
SES	.02	−0.057 to 0.094	.624	−.02	−0.096 to 0.055	.592	−.04	−0.121 to 0.052	.433

Significant *p*-values  < .05 are in bold.

### 3.3. Gender differences in associations

The relationship between anxiety and headache, and depression and headache (but not musculoskeletal pain and abdominal pain) evinced different patterns for boys and girls; neither depression (*β* = .13, *p* = .18) nor anxiety (*β* = .02, *p* = .79) correlated with headache among boys, whereas depression (*β* = .18, *p* = .03), but not anxiety (*β* = −.07, *p* = .22), was related to headache among girls. Even so, the Satorra-Bentler revealed no significant gender differences of the associations between depression and headache (*p* = .84).

## 4. Discussion

In this large-scale, community-based study of Norwegian 10-year-olds, we aimed to determine the unique associations between symptoms of anxiety disorders and pain, and between symptoms of major depressive disorder and pain. Pertaining to our research questions we explored whether pain is (1) associated with depression and/or anxiety, and whether the associations differ according to (2) pain localizations and/or (3) gender. The present results revealed that higher levels of depression—but not anxiety—was related to more musculoskeletal pain and headache. However, the associations between pain and depression were not significantly stronger than those involving anxiety. Moreover, although an association between headache and depression was seen in girls—but not boys, this gender difference did not reach significance.

In the current investigation, results revealed that musculoskeletal pain and headache was associated with depression. Similar patterns of association between musculoskeletal pain, headache and depression have been reported by others who have ruled out common symptoms (i.e., having difficulties concentrating, sleep problems, and fatigue) of anxiety and depression (26, 27). However, we did not find any association of pain to anxiety, which deviates from the findings of Egger, Angold (26) and Egger, Costello (27) who reported that among girls musculoskeletal pain, headache and stomachache was also associated with anxiety. The relatively low effect sizes in the relationship between symptoms of anxiety and depression and recurrent pain across the three locations is perhaps the most notable result reported here, as other studies have shown stronger relationships in populations with mental distress (20) as well as in community samples (2, 15, 17). Of note, in several of the previous studies the children/youth have been providing the data on their emotional problems and pain by responding to questionnaires (15, 17, 20), or by the use of Audio Computer-Assisted Self-Interviewing (ACASI) (13). Self-report bias caused by a common rater may result in artificial covariance between pain and emotional symptoms—increasing the likelihood of spurious or inflated relationships (37). In contrast to studies that rely on reports from the children only, we applied semi-structured interviews with both parents and children to record DSM-4 defined symptoms of anxiety and depressive disorders and a structured interview with children to record pain. Consequently, inflated associations between emotional problems and pain are less likely for the research reported herein.

Although headache and musculoskeletal pain were associated with depression—and not with anxiety—results revealed no significant difference between depression and anxiety in association to pain. Nevertheless, it is worth noting that the results reported here had a *p*-value of 0.056 for headache. With the more pronounced associations between pain and depression in general among these 10-year-old children, it would be of further interest to study the relationship between pain and depression in larger samples while controlling for symptoms of anxiety. Likewise, it would be of interest to study whether depression is more strongly associated with pain compared to anxiety in older children.

A notable finding was that girls were more likely to experience abdominal pain, but neither anxiety nor depression was related to abdominal pain. With the many studies (mainly clinical) reporting a greater likelihood of abdominal pain among depressed, and especially anxious children (25, 38–40), it was unexpected that neither depression nor anxiety were associated with abdominal pain. This could perhaps be due to the effects believed to underlie the association between abdominal pain and psychopathology, such as reduced gut motility or permeability (41) is dependent on higher or more chronic levels of psychological symptoms not evident in the current community sample. Depression predicted headache for girls, but not for boys in the separate models. However, none of the associations between pain location, anxiety and depression was statistically different for boys vs. girls.

## 5. Strengths and limitations

The strengths of this study include the large community-based sample and the use of a semi-structured diagnostic interview to detect symptoms of anxiety and depression—including application of a joint parent-child score of number of symptoms. While community-based samples allow for broader generalization of findings to a general population of children, the use of semi-structured diagnostic interviews ensures discriminant validity between anxiety and depression. However, some limitations should be noted. Of importance, this was a cross-sectional study, thus precluding any causal conclusions. Further, the frequency of pain, anxiety and depression was low. Consequently, statistical power is a potential problem, running a risk of rejecting a false null hypothesis, perhaps specifically concerning abdominal pain which was particularly infrequent. A limitation is also the use of interviews with children only to record pain, relying only on their retrospective memory. Finally, more than 90 percent of mothers or fathers in the study was of Norwegian ethnicity and the sample did not contain great differences in SES. Thus, our findings cannot automatically be generalized to populations of other ethnicities, cultures, or more socioeconomic disadvantage.

## 6. Conclusion

The study investigated associations between anxiety, depression, and pain in three locations (headache, abdominal, and musculoskeletal) in a community sample of Norwegian 10-year-olds. Only depression was associated with two of the three areas of pain; musculoskeletal pain and headache. The associations were not significantly stronger than for anxiety. In the separate models, depression was related to headache for the girls only, but the association was not statistically different compared to the boys. There is a widespread perception in clinical psychology that children routinely express emotions and psychological symptoms through somatic symptoms, in particular abdominal pains and symptoms (21). The effect sizes in this study may indicate lower associations between depression and anxiety and pain in children outside of clinical populations than expected. In particular, it is interesting that our results indicate that the relationship between abdominal pain and psychological symptoms of anxiety and depression is lower in children outside clinical settings than assumed. It should however be noted that the frequency of abdominal pain was low in the present study. Instead, headaches and musculoskeletal pains were associated with depressive symptoms in our sample. These results may, in turn, influence our interpretation of different forms of pain in children, questioning whether less weight should be given to abdominal symptoms as a strong and consistent correlate with psychological problems, compared to for instance headache.

## Data Availability

The datasets generated and/or analyzed during the current study are not publicly available due to restrictions related to participant consent and because the study is still ongoing, but potential collaborators are welcome to contact Lars Wichstrøm, who is the PI of the study.
